# A Model Community Skin Cancer Prevention Project in Maine

**Published:** 2004-03-15

**Authors:** Christine A. Hayden

**Affiliations:** City of Portland, Health and Human Services Department, Public Health Division

The purpose of our program was to create and test a community skin cancer prevention project for replication throughout the state of Maine. The project was a collaborative effort of the Maine Cancer Consortium, American Cancer Society (ACS), and the City of Portland, Health and Human Services Department, Public Health Division. Portland, Me, served as the pilot site.

The National Cancer Institute (NCI) defines skin cancer as a disease in which abnormal cells divide uncontrollably in the outer layers of the skin (1). The American Cancer Society's *Facts and Figures 2001* (the latest year for which these figures are available) estimated that more than 1 million cases of highly curable basal cell or squamous cell cancers would be diagnosed in the United States that year (2). An estimated 9800 U.S. deaths from cancer were projected as well: 7800 from melanoma, the most serious form of skin cancer, and 2000 from other skin cancers. Melanoma was expected to be diagnosed in about 51,400 Americans in 2001. The incidence rate of melanoma has increased about 3% per year on average since 1981. In 2002, NCI announced that researchers showed for the first time that *individual* risk of melanoma is associated with the intensity of sunlight that a person receives over a lifetime (3).

Target audiences for our program were newborns and their parents, children between 5 and 14 years old and their caregivers, and all people living in the Portland area. Protecting skin from excess sun exposure during childhood and adolescence is important in reducing the risk of all types of skin cancer during adulthood. From our anecdotal evidence, many parents of newborns are unaware that sunscreen is not recommended for babies under 6 months of age, and they need better information about how to protect their newborns from the sun. Teaching children and their caregivers to follow ACS guidelines will help protect their skin for years to come. It will also help children to develop healthy habits they can maintain throughout their lives.

Our skin cancer prevention project, which took place during 2002, mirrored the goals of *Healthy Maine 2010* to increase the proportion of people who limit sun exposure and use protection when exposed to sun (4). Our specific objectives were the following:

1. Increase the proportion of new parents who are aware of the dangers of sun exposure to newborns and the proper ways to protect their babies from sun exposure.
2. Improve sun protection policies for the 700 youths participating in Portland's Parks and Recreation summer camp program.
3. Increase community awareness of the dangers of unprotected sun exposure.

To help us design and implement our action plan, in fall 2001 we recruited members for a sun protection team from among our group of partners and from the ACS Cancer Control Advisory Council. The team consisted of one staff person from the City of Portland's Public Health Division, 2 staff members from the ACS, nurses from the 2 participating hospitals, staff from the City of Portland's Parks and Recreation Department, and staff of the Sea Dogs, Portland's minor league baseball team. Not all staff members were involved with all components of the program.

The project was funded through the ACS for $10,000 for a one-year period. One City of Portland staff member was paid for approximately 3 hours of work per week to coordinate the program. In addition, approximately $3500 was spent on supplies, $2000 for advertising and brochures, and $300 for educational sessions.

Our skin cancer prevention project consisted of 3 major components, which are described below.

## No Sun for Baby

This hospital-based initiative targeted parents of babies born during May (Skin Cancer Awareness Month) 2002. Of the 3 hospitals in Portland, 2 are known for their maternity or birthing units: Maine Medical Center and Mercy Hospital. We contacted the nurse managers of the maternity unit at Maine Medical Center and the birthing unit at Mercy Hospital to discuss and obtain approval for our plans and were told that a total of between 350 and 400 babies are typically born during May at these 2 hospitals. ACS staff delivered *No Sun for Baby* kits to each hospital unit. Each kit included a beach pail and shovel with a *Slip! Slop! Slap! Wrap!* message as well as a baby sun hat and brochure from the Skin Cancer Foundation. *Slip! Slop! Slap! Wrap!* means "Slip on a shirt, slop on sunscreen with an SPF of 15 or higher, slap on a hat with a wide brim, and wrap on sunglasses" (5). Both hospitals agreed to promote sun safety information by distributing brochures and/or by talking about Skin Cancer Foundation recommendations and/or ACS guidelines in childbirth education classes, Lamaze classes, parenting classes, and discharge packages. An evaluation postcard was also included in the kit for parents to fill out and return to the ACS. We distributed 380 *No Sun for Baby* kits, and both hospitals have committed to incorporating sun safety messages in childbirth education classes on a continuing basis.

## Parks and Recreation Sun Protection Guidelines

In working with the team member from the City of Portland's Parks and Recreation summer program, we learned that there were no sun protection policies or guidelines for the approximately 700 campers aged 5 to 14 years who participate annually. For the 2002 summer camp season, we distributed sun protection guidelines for outdoor recreation or work and provided technical assistance to Parks and Recreation management on adapting the guidelines to meet their needs (6). We conducted educational sessions for 50 Parks and Recreation staff members and gave them *Slip! Slop! Slap! Wrap!* educational brochures. We also discussed the rationale for having and enforcing guidelines. Enforcing the guidelines would prove to be difficult because some children arrived at camp without shirts, sunscreen, hats, sunglasses, or other sun protection. In the educational sessions with counselors, we stressed the importance of counselors providing positive role models for campers. We encouraged counselors to use the *Slip! Slop! Slap! Wrap!* model. We also emphasized the importance of creating shady areas for campers. As a result, Parks and Recreation management purchased portable tents for each campsite and created shade structures at the municipal pools.

All parents of campers were provided a copy of the sun safety guidelines as well as a copy of the *Slip! Slop! Slap! Wrap!* educational brochure. The Parks and Recreation Department received only one phone call during the summer from a parent of a child who had been sunburned while at camp.

## Protect the Skin You're In Day

We distributed 500 sunscreen samples and *Slip! Slop! Slap! Wrap!* educational brochures to approximately 6200 baseball game attendees and 200 staff in collaboration with Portland's minor league baseball team, the Sea Dogs, on June 9, 2002. Slugger, the Sea Dogs mascot, performed the *Slip! Slop! Slap! Rap!* throughout the game. Game announcers read *Slip! Slop! Slap! Wrap!* messages (Figure) between innings, and we held drawings for T-shirts donated by the ACS. The Sea Dogs donated space in their program for a half-page *Protect the Skin You're In* advertisement. The number of programs distributed during summer 2002 was 22,750. In addition, Sea Dogs management agreed to sponsor an annual *Protect the Skin You're In Day*.

**Figure. Public service announcements, Portland Sea Dogs baseball game, community skin cancer prevention project, Portland, Me, June 9, 2002.**

Unprotected skin can burn within 12 minutes. Slip on a shirt! Slop on sunscreen! Slap on a hat! Wrap on sunglasses!About 80% of skin cancers could be prevented by protection of skin from the sun's rays.Skin cancer is the most common form of cancer — there are more than 1.3 million diagnoses each year.To protect your skin from the sun's rays, apply sunscreen with SPF 15 or higher every 2 hours.Sunscreen is not recommended for children under the age of 6 months — they should be shaded from the sun.Sunlamps and tanning booths are as harmful to your skin as the sun.It is most important to protect your skin between 10:00 AM and 4:00 PM, when the sun's rays are most intense.Protect your children from sunburns! Research shows a link between sunburns in children and an increased risk of melanoma and other skin cancers later in life.

All components of the Portland sun protection pilot program could easily be replicated and sustained by other communities. The cost for each component is low. Several steps can decrease costs. For example, hospital volunteers could make sun hats for the *No Sun for Baby* program. Hospitals could also solicit merchants for donated pails and shovels. Many different kinds of organizations can participate: hospitals, health agencies, municipalities, sports teams, private and public camps, summer camps, YWCAs and YMCAs, Boys & Girls Clubs of America, scouting organizations, and others.

Buy-in from each organization is essential to program success. We often found that individuals within a partner organization who had friends or relatives with sunburns, suspicious moles, or skin cancer itself would often take leadership roles within the organization.

This program offers tremendous news media opportunities for promoting sun protection messages. We contacted local television stations to publicize the *No Sun for Baby* program. Our staff members also happened to know parents who were expecting babies in May 2002. These prospective parents agreed to be interviewed by the news media, and an interview was aired on *Health Beat*, a segment of a local television evening news program.

We also exhibited our program at poster sessions at the 17th National Conference on Chronic Disease Prevention and Control in St. Louis, Mo, February 17–19, 2003, and the Centers for Disease Control and Prevention Cancer Conference in Atlanta, Ga, September 15–18, 2003. This gave us the opportunity to share our work with other organizations throughout the country so they could replicate it within their own communities.
